# Erythrocytes Functionality in SARS-CoV-2 Infection: Potential Link with Alzheimer’s Disease

**DOI:** 10.3390/ijms24065739

**Published:** 2023-03-17

**Authors:** Elena Kosenko, Lyudmila Tikhonova, Gubidat Alilova, Carmina Montoliu

**Affiliations:** 1 Institute of Theoretical and Experimental Biophysics of Russian Academy of Sciences, 142290 Pushchino, Russia; 2 Hospital Clinico Research Foundation, INCLIVA Health Research Institute, 46010 Valencia, Spain; 3 Pathology Department, Faculty of Medicine, University of Valencia, 46010 Valencia, Spain

**Keywords:** SARS-CoV-2, COVID-19, Alzheimer’s disease, erythrocyte metabolism, morphological changes, oxidative stress, erythrocyte dysfunction

## Abstract

Coronavirus disease 2019 (COVID-19) is a rapidly spreading acute respiratory infection caused by SARS-CoV-2. The pathogenesis of the disease remains unclear. Recently, several hypotheses have emerged to explain the mechanism of interaction between SARS-CoV-2 and erythrocytes, and its negative effect on the oxygen-transport function that depends on erythrocyte metabolism, which is responsible for hemoglobin-oxygen affinity (Hb-O_2_ affinity). In clinical settings, the modulators of the Hb-O_2_ affinity are not currently measured to assess tissue oxygenation, thereby providing inadequate evaluation of erythrocyte dysfunction in the integrated oxygen-transport system. To discover more about hypoxemia/hypoxia in COVID-19 patients, this review highlights the need for further investigation of the relationship between biochemical aberrations in erythrocytes and oxygen-transport efficiency. Furthermore, patients with severe COVID-19 experience symptoms similar to Alzheimer’s, suggesting that their brains have been altered in ways that increase the likelihood of Alzheimer’s. Mindful of the partly assessed role of structural, metabolic abnormalities that underlie erythrocyte dysfunction in the pathophysiology of Alzheimer’s disease (AD), we further summarize the available data showing that COVID-19 neurocognitive impairments most probably share similar patterns with known mechanisms of brain dysfunctions in AD. Identification of parameters responsible for erythrocyte function that vary under SARS-CoV-2 may contribute to the search for additional components of progressive and irreversible failure in the integrated oxygen-transport system leading to tissue hypoperfusion. This is particularly relevant for the older generation who experience age-related disorders of erythrocyte metabolism and are prone to AD, and provide an opportunity for new personalized therapies to control this deadly infection.

## 1. Introduction

Coronavirus disease 2019 (COVID-19) is an acute infection of the respiratory tract caused by the newly discovered virus SARS-CoV-2 [1]. At present, the disease pathogenesis remains poorly understood and there are no specific antiviral drugs with therapeutic potential for the treatment of infected patients. According to the data available, the main difference between SARS-CoV-2 infection and infections with any other known previously human coronaviruses is that the former often remains asymptomatic [2], but can lead to multiple organ failure if it spreads in conjunction with acute respiratory distress syndrome (ARDS) [3,4], with increased mortality among older people [5]. There are many causes of multiple organ failure, but the most important is that at the earliest stage of infection the virus simultaneously attacks many host cells with angiotensin converting enzyme 2 (ACE2) receptors [6], including cells of the respiratory and gastrointestinal tract, liver, heart, kidneys, central nervous system, endothelium of the blood vessels, oral mucosa, and immune cells [7].

Moreover, many different pieces of data show that immune response to the viral infection may result in excessive cytokine release, evolving into inflammatory cytokine storm that can lead to multiple organ dysfunction and become life threatening [8,9,10,11,12,13,14]. Although there is sufficient evidence to indicate that multiple organ failure is not only associated with COVID-19 [15] but is also apparently the main hallmark of disease severity, the pathogenetic mechanisms by which damage to tissues and organs occurs are still not completely understood. In this connection, based on several hypotheses regarding various mechanisms of interaction between SARS-CoV-2 and erythrocytes (red blood cells, RBCs) [16,17], it is assumed that extremely restricted oxygen supply by disturbed RBCs may be a root in the development of hypoxia-induced multiple organ injury and the main cause of high mortality of critically ill COVID-19 patients [17,18,19,20,21].

Hemoglobin (Hb) is the dominant protein in RBCs, the functions of which are to bind, transport, and unload oxygen to tissues, in a manner dependent on erythrocyte metabolism [22,23] which is responsible for hemoglobin–oxygen affinity (Hb-O_2_ affinity). However, the efficacy of oxygen delivery by RBCs as one of the components in the integrated gas-transport system has rarely been assessed in combination with the other indicators usually measured in a clinical setting [24], in order to detect the limit of O_2_ transport reach. This leads in turn to erroneous conclusions regarding the establishment of the true reasons for the development of tissue hypoxia and detection of regional perfusion abnormalities [25,26,27,28,29], thereby failing to improve the process of therapeutic assistance.

Certainly, tissue oxygenation depends not only on RBCs and Hb function, but also on the inter-dependent interplay between the respiratory and cardiovascular systems involved in oxygen transport [30,31,32]. However, despite the importance of these major systems, the final stages of oxygen delivery to tissues where gas exchange occurs are carried out exclusively by RBCs that must be functionally active and, consequently, absolutely “healthy” [33]. Therefore, when factors determining oxygen binding, transport and delivery are identified, clinicians should also focus on the indices of metabolic abnormalities of RBCs [30,34,35]. Quantitative assessment of these parameters in patients with COVID-19 has already begun, but the data from different researchers are not only contradictory [24,36,37,38,39,40] but have caused heated debate [41,42,43], and the question remains open of whether global abnormalities in erythrocyte composition found in infected patients [20] are a contributing factor to the pathophysiology of tissue hypoxia caused by SARS-CoV-2, and further research and discussion are needed (discussed below).

In light of the foregoing, this review presents emerging evidence supporting the need for more knowledge about the relationship between biochemical aberrations and the efficacy of oxygen transport by RBCs as an additional cause of progressive and irreversible failure in the integrated oxygen-transport system. This will help minimize inaccurate and even erroneous conclusions for patients, especially the older generation with age-related disorders of RBC metabolism who are prone to AD, as well as individuals with acquired changes in Hb function and those with Hb congenital anomalies [25,26,44,45].

We also briefly review potential causes for the discrepancies between the available data and certain difficulties in analyzing Hb-O_2_ affinity changes in laboratory and clinical conditions. Meanwhile, there are emerging reports that patients with severe COVID-19 experience various Alzheimer’s disease (AD)-related symptoms ranging from depression and agnosia to hypometabolic/hypoxic encephalopathy and dementia [46,47,48,49,50,51,52,53], suggesting that their brains have been changed in ways that increase the risk of developing the sporadic (non-genetic) form of AD, regardless of age [54]. However, it should be noted that the sporadic form of AD is recognized as multifactorial disorder [55], and debates continue regarding what pathological factors trigger the development of the disease.

Cell viability in the brain and the organ’s myriad functions are critically dependent on an uninterrupted delivery of oxygen by the blood, and even a short pause in the oxygen supply to the brain causes its damage [56]. Chronic oxygen deficiency eventually leads to irreversible brain pathology and the further advancement of permanent cognitive impairment [57,58,59], and apparently plausible vascular/erythrocytic hypotheses [60,61] attribute these findings to hypoperfusion and disturbed RBC metabolism and function, underlying the development of energy crises in the brain, neurodegeneration, and cognitive deficit [56,58,59,62,63,64,65,66,67]. According to data on the role of structural metabolic abnormalities underlying functional deficits of RBCs in AD pathophysiology [33,61,67,68,69,70,71,72,73,74,75,76], we further summarize the available knowledge indicating the possible uniformity of COVID-19 neurocognitive impairments in the context of known mechanisms of brain dysfunction in AD.

## 2. Intracellular RBC Metabolism, Allosteric Effectors, and Oxygen-Transport Function

RBCs are specialized cells that carry and release oxygen to the tissues for supporting aerobic energy metabolism, coupled with the synthesis of ATP in mitochondria. However, the oxygen-transport function of RBCs is often viewed in a simplified way and RBCs themselves are perceived merely as small bags filled with hemoglobin, that easily bind oxygen in the lungs and just as easily release oxygen to tissues. Meanwhile, the presence of oxygen in red blood cells is the main condition, but not the only requirement. It is generally accepted that the capacity of RBCs for binding, transport, and delivery of oxygen to tissues depends on heterotropic allosteric modulators, formed as a result of metabolic processes in membranes and inside RBCs [22,23], which in addition to hemoglobin oxygen affinity [77], regulate NO-dependent hypoxic vasodilation [78] and tissue perfusion [79,80], and preserve ion and systemic metabolic homeostasis [81].

Interestingly, RBC indices commonly measured in clinical settings, such as number of circulating RBCs, erythrocyte sedimentation rate (ESR), hemoglobin count, hematocrit test, and others, including their correct interpretation based on accepted reference values, are widely considered to play a crucial role in assessing health status and clarifying accurate diagnosis of disease [82,83]. Meanwhile, in parallel with this diagnostic, no estimate is usually given on how changes in the results of these hematology clinical laboratory tests outside the accepted reference ranges (for example, a sharp increase in ESR) affect the oxygen-transport function of RBCs.

It should furthermore be noted that RBCs are cells that “work” even under physiological conditions, in an environment that is not always comfortable. In order to resist harmful factors that occur both in the bloodstream and inside the cells [84] and may impair RBC function and promote cell death, the RBCs should be “healthy” with intact membranes and no changes in intracellular metabolic pathways [22,77,85].

Indeed, the formation of superoxide radicals constantly takes place in RBCs [86] during oxygen binding to hemoglobin in the lungs. In kidneys, when the cells are surrounded by a hypertonic environment, they experience osmotic shock. In tight capillaries where gas exchange occurs, RBCs are exposed to extensive mechanical stress [87].

However, it is recognized that under physiological conditions these factors seem to affect the function and survival of only senescent or damaged RBCs, while most populations of young and middle-aged cells are more resistant and remain “healthy” despite the constant destructive impact [88].

What are the defense mechanisms that ensure the protection of circulating RBCs and support their viability and gas-transport function?

Primarily, glycolysis and salvage pathways provide energy in the form of ATP [89]. The pentose phosphate shunt is required for NADPH production, which together with antioxidant enzymes, i.e., glutathione reductase and glutathione peroxidase, converts oxidized glutathione to reduced glutathione, a major RBC antioxidant which is important for protection against oxidative damage and maintains hemoglobin in a reduced, active form [90]. RBCs also exhibit fairly high superoxide dismutase activity, which maintains a safe level of the superoxide radical (O_2_^•−^) that is constantly formed by Hb autoxidation during oxygen binding to hemoglobin in the lungs [84,86]. Catalase and peroxiredoxin-2-peroxidase are the other antioxidant enzymes that collectively destroy hydrogen peroxide, preventing vital biomolecules from its damaging effect and, in particular, protecting Hb from oxidative destruction [88]. In addition, important supporting processes in RBCs include the NADH-cytochrome b5 reductase pathway reduces methemoglobin (metHb) that is incapable of transporting oxygen [91], and also xenobiotic detoxification by the enzyme glutathione transferase [92]. Furthermore, the proteasome machinery plays an important role in protecting the erythrocyte membrane and is involved in the degradation of oxidized Hb during membrane damage [93,94]. In the Rapoport–Luebering shunt [77], 2,3-diphosphoglycerate (2,3-DPG) is produced, which is the main allosteric modifier of Hb-O_2_ affinity [95]. The shunt activity is regulated by many factors, especially by the rate of glycolysis and the activities of two enzymes of non-glycolytic pathways, diphosphoglycerate mutase (DPGM) and diphosphoglycerate phosphatase (DPGP) [28]. Thus, the metabolism of RBCs is represented by multiple, interconnected systems consisting of soluble, structural proteins, carbohydrates, lipids, anions, cations, cofactors, metabolites, and antioxidants in particular amounts. If a change of at least one component in the integrated system of metabolic pathways occurs under pathological conditions, this leads at once to the loss of RBC’s integrity and functional capacity [77], and even to cell lysis directly into the bloodstream. This is extremely dangerous, since it may lead to hypoxia [96,97] and adverse outcomes following free Hb-iron-dependent cellular oxidative damage and progression of multiple organ failure, which are included in the etiology and pathophysiology of various diseases, including Alzheimer’s disease [98,99,100,101,102,103].

Therefore, in order to assess the efficiency of oxygen transfer from the lungs to the tissue of coronavirus-infected patients with hypoxemic respiratory failure [104], it is important to calculate not only how much oxygen is available in the blood in total (arterial oxygen saturation—SaO_2_, and partial pressure of oxygen—PaO_2_), but also to understand whether hemoglobin can bind the maximum possible amount of oxygen in the lungs and release the required amount of O_2_ to the tissue, which is determined by the Hb-O_2_ affinity [21,22,24,36,43,105].

Furthermore, according to the research data nearly 5% of the world’s population are hemoglobinopathy carriers [106] and to date more than 1000 Hb variants characterized by increased or decreased oxygen affinity have been identified in people from diverse ethnic groups [29,107,108]. Although the consequences of many abnormal hemoglobin variants are well known, for instance, anemia, reduced membrane deformability, premature aging and destruction of circulating red blood cells, impaired gas exchange, etc. [108,109,110], these abnormalities have limited clinical applications making it difficult to predict the severity and nature of oxygen deficiency at the tissue level. This can lead to errors when planning therapy, especially mechanical ventilation [110,111] for critically ill patients in whom Hb variants appear in indefinite proportions during infection [109]. Thus, in the presence of hemoglobin with a low affinity for O_2_, leading to a decrease in oxygen–hemoglobin binding in the lungs, it can be expected that prolonged use of mechanical ventilation for critically ill patients with COVID-19 can cause not only worse pulmonary outcomes [112] and respiratory failure [113], but also vasoconstriction, impaired blood flow, and limited O_2_ delivery to organs such as the brain, heart, skeletal muscle, and others [114,115,116]. This may explain the high incidence of multiple organ failure [117] and greater risk of poor outcomes in these patients [118,119,120]. This issue is further complicated by the fact that even patients with normal PaO_2_ or SaO_2_ but in the presence of Hb with high oxygen affinity may still present tissue hypoxia, stemming from a reduced ability to release O_2_ in tissues, and supplemental oxygen may be warranted irrespective of oxygen saturation or arterial oxygen partial pressure [26,109,110,121,122].

Thus, it is clear that there is a need to establish and implement in clinical laboratories a two-sided reference interval of erythrocyte indicators associated with regulation of the oxygen binding capacity of Hb [109,123], depending on gender, genetics, ethnicity, and other biological characteristics [116,124]. The use of these indicators will enable the most appropriate treatment to be selected for each patient.

## 3. Peculiarities of Metabolic Abnormalities of Erythrocytes in Elderly Patients: Forgotten Prognostic Factors for Severity of COVID-19?

Although all age groups are at risk for SARS-CoV-2 infection, a major feature of the infection is increased mortality among older people [125,126,127,128,129,130]. Unfortunately, the underlying mechanisms leading to worse age-related outcomes and increased mortality risk in the older age group are not fully understood. Numerous factors have been identified as predictors of poor outcomes, such as chronic comorbidities (hypertension, cardiovascular disease, diabetes [131,132,133], inadequate immune system response, mitochondrial dysfunction [134], faster virus replication and reproduction [135], Alzheimer’s disease and related dementia [136], and so forth [137]).

Moreover, it should be kept in mind that the intracellular metabolism of RBCs undergoes stable and permanent changes in older individuals [61,72,138,139,140], which under the influence of pathological factors may constitute an additional risk factor for RBC dysfunction [33]. Indeed, the RBCs of the aged persons are characterized by a reduced rate of glycolysis, pentose phosphate pathway, and cation transport, confirmed by reduced activity of all glycolytic enzymes, reduced glucose uptake rate [61,72,141,142], the formation of pyruvate and lactate, ATP concentration [143], and sharply reduced Na^+^,K^+^-ATP-ase [144], compared with younger individuals [145]. Additionally, RBC abnormalities in older subjects were associated with disturbed calcium homeostasis, higher levels of calpains, caspases-3, increased proteolysis [139,146], hydroperoxide generation, and decreased antioxidant enzyme activity [61,142,147,148,149] and circulatory lifespan [88,139,150,151]. The enzyme activity of the Rapoport–Luebering shunt, DPGM, and DPGP, which maintain stable levels of 2,3-DPG, the main regulator of Hb-O_2_ affinity, decreases simultaneously with a significant decrease in 2,3-DPG concentration in RBCs in the elderly [142,152,153], resulting in violation of the allosteric properties of hemoglobin [154]. Together with the currently available information, these results indicate that oxidative stress is already present to some extent in RBCs in the elderly [88,155,156,157].

Increased blood viscosity, RBC aggregation [158], modification of the band 3 protein [147], impaired blood rheology [159], and disruption of the RBC transport function [22,58,88,160] are common signs of physiological aging. It is clear that with the appearance of additional adverse factors that can interact with RBCs in the bloodstream, the propensity of already compromised cells in older subjects for disease and life-threatening hypoxemia/hypoxia increases significantly [33,61,72,142,145,160,161,162].

Thus, assessment of the biochemical diversity of RBCs in patients of different age groups, inevitably leading to different levels of adaptive response to hypoxic conditions [163,164], is useful for predicting the severity of COVID-19 and selecting individual therapies. Moreover, there is a need to identify potential reasons for inconsistent experimental results and the existing opposing views about the role of RBCs in disrupting convective oxygen transport in SARS-CoV-2 infection [20,40,165], associated not only with the patient’s age but also with the use of nonunified experimental design that does not permit the researcher to consider a myriad of variables affecting the results.

## 4. Potential Causes of Discrepancies in Available Data and Challenges in Analyzing Changes in the Affinity of O_2_ for Hb in Laboratory and Clinical Settings

It is common knowledge that internal Hb-O_2_ affinity in vivo is very high [166], meaning that allosteric effectors reducing the affinity of Hb-O_2_ must be continuously formed in RBCs. In the absence of these effectors, high Hb-O_2_ affinity improves oxygen loading in the lungs but impairs oxygen unloading at the tissue level. As mentioned above, the glycolytic intermediate 2,3-DPG, quantitatively the most important organic phosphate produced within RBCs [167,168], is one of the main allosteric ligands promoting oxygen offloading in the tissues by lowering Hb-O_2_ affinity [22] and plays a prime role in modulating the ability of the whole blood to transport oxygen. The reduction in Hb-O_2_ affinity, associated with RBC metabolism through 2,3-DPG with an oxygen-transport function, is a complex and tightly regulated process [123] where the quantitative relationship between the concentration of 2,3-DPG and oxygen release is very precise [123,169,170,171,172]. Its violation leads to increased risk of adverse outcomes in many conditions associated with hypoxemia/hypoxia, as has been repeatedly proven [77,173,174,175,176,177,178,179,180,181].

In clinical settings, however, Hb-O_2_ affinity is routinely measured by the oxygen dissociation curve (ODC), a graphical representation of the relationship between oxygen saturation and oxygen partial pressure, where p50 (the oxygen tension when Hb is 50% saturated with oxygen) is used as the sole descriptor of Hb-O_2_ affinity, usually calculated under standard conditions (pH: 7.4, PCO_2_: 40 mmHg, temperature: 37 °C) [36,41,182,183], i.e., in the “artificial normalization” of a potentially very abnormal blood-flow environment in a seriously ill patient, leading to the elimination of many influencing factors that can change the ODC unpredictably [43], thereby leading to conflicting results [184,185]. Another bottleneck in most previous investigations of Hb-O_2_ affinity was that the concentration of 2,3-DPG in RBCs of patients with SARS-CoV-2 infection was not usually measured [41], and the concentrations of Hb, phosphate, age (aging red cells or patients), history of heart failure [39], or a fixed value of 2,3-DPG [186,187] were used as a sensitive index in predicting 2,3-DPG concentration. However, the steady-state concentration of 2,3-DPG in RBCs varies widely among patients with the same disease, and does not always correlate with P50, Hb concentration, or plasma phosphate [188,189,190,191]. Therefore, in routine clinical practice it would be desirable to add the measurement of RBC 2,3-DPG concentration, together with identification of the endogenous factors influencing its concentration and thus influencing ODC/Hb-O_2_ affinity [36].

Furthermore, some patients may have chronic diseases [192,193] and permanently receive drugs such as different types of hormones [170,194,195,196], β-adrenoblockers [197], anesthetics [198], hydroxychloroquine [39,199], salicylates [200], and others [192] which might influence the ODC. Surprisingly, articles on the measurement of ODC/Hb-O_2_ affinity in patients with COVID-19 have been published without this information [41]. Therefore, it can be assumed that the absence of a more complete set of factors affecting the position of the ODC can lead to either under- or over-estimation of true Hb-O_2_ affinity, particularly considering the relatively small numbers of patients in each study and poor matches between patients and controls in terms of gender, age, etc., and the lack of a unified experimental design for evaluating the ODC and calculating P50 [41,43]. This is especially the case in COVID-19 patients with severe anemia and hypoxia, reflecting to a limited extent (if at all) the impaired oxygen-transport function of RBCs [201].

In practice, mixed findings for Hb-O_2_ affinity have been obtained in COVID-19 patients. Thus, no change of Hb-O_2_ affinity was revealed in some investigations [37,38,39,40,202], while in others the researchers reported increases [36,186] or decreases in this index [20], suggesting the need to measure additional markers relating to tissue oxygen perfusion. For example, in some COVID-19 patients, the appearance of rigid RBCs, including mushroom-shaped RBCs [203], schizocytes [204], and sphero-echinocytes with irreversible metabolic, structural changes [205] and phosphatidylserine inversion [206] in the bloodstream points to an alteration in membrane charge [207], structural membrane damage [20], and decreased turnover rate [208]. Altogether, this can dramatically increase aggregation [209] and reduce microvascular perfusion [210,211], eventually affecting the efficiency of oxygen transport [212,213]. Therefore, the analysis of changes in the morphology and appearance of rigid cells in the bloodstream of the COVID-19 patient can enrich knowledge of the progression of impaired tissue perfusion. However, the metabolic and morphometric parameters of RBCs can vary significantly depending on the experimental conditions [207,214], and the use of a wide range of methods may often lead to inconsistency between measured indicators and those obtained in vivo, leading to difficulties in their interpretation.

According to the recommendations of the International Committee for Standardization in Haematology [215], structural and functional RBC characteristics should be studied using highly purified cells obtained by the fractionating method [216] from blood collected in a uniform manner and previously purified from leukocytes and platelets [217], using sensitive, quantitative methods that are specific for RBCs [215,218,219]. Concurrently, consideration should also be given to the plausible effects of circadian rhythm [220], patient hydration status [221], physical activity [222], recent fluid and food intake [223], recent blood transfusions [224], drugs [225], ambient temperature [226], humidity, sample collection and storage procedures [227,228], selection of an anticoagulant [229], cell washing, resuspension, and lysis buffering [230], and many other factors described in new guidelines for hemorheological laboratory techniques [231] and by experts from the World Health Organization [232]. The crucial importance of such an approach is emphasized by the fact that neither biologists nor clinicians fully take into account the influence of many factors, and therefore sometimes use RBC data that do not necessarily reflect the in vivo state characteristic of a particular pathology [221]. This leads to conflicting results and conclusions, and limits the understanding of the etiological role of RBCs as one of the components of a complex oxygen-transport system involved in the development of life-threatening hypoxemia/hypoxia and multiple organ dysfunction during infection [233]. To avoid this, and to improve the effectiveness of treatment for COVID-19 patients and turn this deadly disease into a well-controlled infection, the current practice should obviously be changed as soon as possible. Understanding the role of RBCs in COVID-19 can assist the choice of treatment and therapies against this infection, and is critical for the success of continued control of infectious diseases worldwide.

## 5. Erythrocyte Metabolic Disturbances as an Indicator of Mental Disorders: A Possible Link between SARS-CoV-2 Infection and Alzheimer’s Disease

To maintain cell viability and a myriad of highly energy-demanding functions, the brain the highest level of oxidative metabolism compared with the other organs, producing the majority of ATP [57,234,235,236] as a source of energy. Paradoxically, the brain has limited reserves of oxygen [237], and therefore requires a continuous supply of oxygenated blood. As a result, irregular oxygen delivery to the brain for even a short period causes brain damage [235] leading to a loss of consciousness, and chronic oxygen deficiency (regardless of the etiology of the disease) can result in hypoperfusion and irreversible brain damage, thereby provoking encephalopathy with clinical symptoms ranging from minimal changes in intellectual function to dementia and coma, often fatal outcomes [59,238,239,240,241,242,243,244], suggesting some common biochemical background for neurological signs in different diseases.

In recent years, advanced research into RBCs isolated from the blood of patients suffering from various diseases has revealed highly significant positive correlations between numerous biochemical and morphological aberrations in RBCs and the development of various types of dementia syndromes [70,74,75,76,162,245,246,247,248,249]. In particular, it became possible to formulate the vascular/erythrocytic hypothesis of the development of energy crisis in the brain, neurodegeneration, and cognitive deficit at onset of AD. These disorders are considered to be stimulated by hypoperfusion and impaired RBC metabolism and function limiting the continuous supply of oxygen to the brain [33,61,67,162,250,251,252,253]. SARS-CoV-2 infection is no exception. Considering the importance of attitudes towards the development of oxygen deficiency in the brain, the identification and quantification of RBC parameters is instrumental in gaining insight into the reasons for the development of AD-like neurological symptoms in patients with severe COVID-19, at any age [49,54,254,255,256].

Available metabolomics data indicate that RBCs from COVID-19 patients had significantly enhanced glycolysis rates compared with controls, as evidenced by both the accumulation of ATP and many other glycolytic intermediates and by high levels of rate-limiting enzymes of glycolysis. Levels of 2,3-DPG, the main allosteric modifier of Hb-O_2_ affinity, were also significantly increased in RBCs from COVID-19 patients [20]. The authors concluded that “increases in RBC glycolytic metabolites are consistent with a theoretically improved capacity of hemoglobin to off-load oxygen as a function of allosteric modulation by high-energy phosphate compounds, perhaps to counteract COVID-19-induced hypoxia” [20].

Indeed, there is strong evidence that increases in 2,3-DPG and ATP concentration (decreased Hb-O_2_ affinity) in circulating RBCs, with simultaneous significantly increased rates of glucose consumption, are among the main compensatory mechanisms that arise sometimes quite rapidly [257] to improve O_2_ delivery to tissues under grossly inadequate conditions [123], for example, at high altitudes [175] or in patients with hypoxemic/hypoxic diseases such as anemia and cardiopulmonary insufficiency [22,258,259,260,261]. It is apparent that a similar compensatory mechanism can also occur during SARS-CoV-2 infection, the main pathophysiological consequences of which are progressive hypoxia [262] and hypoxia-related multiorgan pathology [263,264].

However, the authors of the above article [20] also found that SARS-CoV-2 infection is associated with impaired structural membrane homeostasis at the level of cytoskeleton proteins and membrane lipids, as well as with a “higher degree of oxidative stress” in patients’ RBCs [20]. It is therefore impossible to assume that COVID-19 patients have completely overcome their difficulties with oxygen transport, or that 2,3-DPG-adaptive response of RBCs would be enough to protect tissues against damage following hypoxia.

It is generally accepted that clear signs indicating disturbances in erythrocyte membranes underlie the loss of specific biconcave shape, deformability, and progressive loss of membrane surface area in cells [219,265,266,267,268,269,270,271,272], which in combination with oxidative stress [273], vesiculation of cells [274], and significant systemic microcirculatory dysfunction [209] may dramatically affect oxygen delivery to tissues in patients with almost any diseases [275].

Given this causal relationship, it can be suggested that the reduced Hb-O_2_ affinity (increased 2,3-DPG) found by the authors in RBCs of COVID-19 patients [20] could allow oxygen to freely detach from Hb at the site of gas exchange. However, it remains unclear how the non-deformable cells found in COVID-19 patients [209,276], with abnormal morphology [203,277] and oxidative stress [278] leading to capillary occlusion [279] and impaired RBC flow [85,88,278], are able not only to counteract COVID-19-induced hypoxia, but even to reach the sites of gas exchange [280]. Therefore, the question arises as to how nondeformable RBCs overwhelmed by oxidant stress are able to traverse capillary beds that have diameters less than those of RBCs [279], engage in gas exchange, and then restore the normal their shapes for further travel.

In addition, the combined effect of the destruction of membrane structures and the presence of a higher degree of oxidative stress in the RBCs of patients with COVID-19 [20], leads to the formation of metHb [281,282,283,284,285,286], unable to carry oxygen [88,90,287,288] and thereby the initiator of encephalopathy, the clinical manifestations of which (depending on the degree of metHb accumulation) [289] can vary from asymptomatic to severe hypoxia that does not respond to supplemental oxygen therapy, leading to changes in mental status, coma, and death [290,291,292].

**Figure 1:**
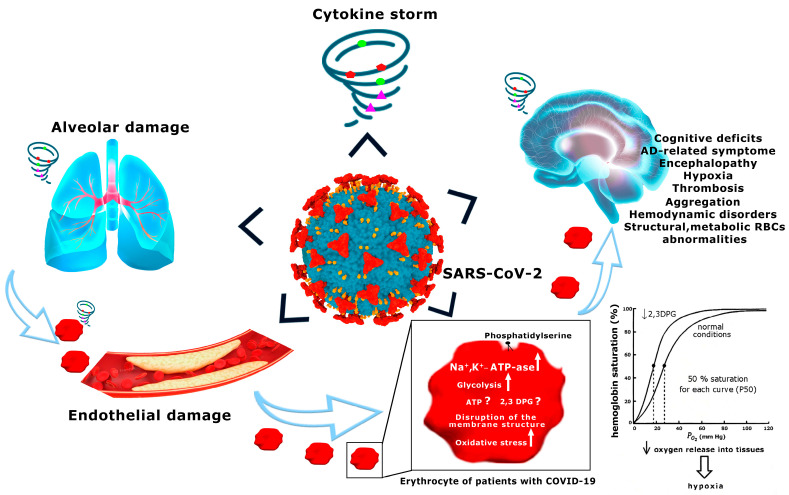
Multifactorial pathogenesis of COVID-19-related neurological complications includes hyperinflammation and cytokine storm, resulting in structural and metabolic RBC abnormalities, hemodynamic disorders, aggregation, thrombosis, impairment of the oxygen-transport function, hypoxia, encephalopathy, AD-related symptoms, and cognitive deficits.

It is quite obvious that the RBCs are highly susceptible to SARS-CoV-2-induced injury [293,294,295,296], and can provide early warning signals of abnormalities of tissue oxygenation [19,297], which in AD can lead to neurological symptoms [33,72,161,162,298,299] and complications of the disease (Figure 1). However, lack of sufficient available data on RBCs of COVID-19 patients, and, moreover, misleading interpretations of these data, do not allow for full-scale comparison of the changes occurring in RBCs in COVID-19 and AD. In this case, it is only possible to point out some similarities.

Firstly, global membrane defects [300,301], proteolysis [302], strong impairment of blood flow, deformability, and shape [248], and oxidative stress [145,303,304] are the main pathological changes of RBCs in AD patients [145,148,305], which are identical to those found in patients with COVID-19 [203,209,276,293].

Secondly, in addition to these structural and biochemical abnormalities, our recent work also indicates that the values of all parameters of the glycolytic system are significantly higher in RBCs of AD patients than those obtained from age-matched controls, indicating an acceleration of the RBC glycolytic pathway [142,148], as in COVID-19 [20].

The exception was that the concentrations of ATP and 2,3-DPG would be significantly reduced in patients with AD [148,153] compared with the control, while in patients with COVID-19, the levels of these metabolites in RBSs compared with the control would be increased, as stated above [20]. The exact causes of this inconsistency are not clear and may be multifactorial.

One of the factors that very probably contributes to altered energy metabolism in the RBCs of AD patients is the enhanced entry of Na^+^ into the cells [306,307]. Activation of glycolysis in the RBCs of AD patients can apparently be considered an adaptive process aimed at maintaining the ATP concentration necessary for up-regulation of Na^+^,K^+^-ATP-ase, which in turn maintains ionic homeostasis in cells. Indeed, the activity of Na^+^,K^+^-ATP-ase in the RBCs of AD patients was increased compared with that found in age-matched controls [61,153,308]. As already noted, this increase in enzyme activity was accompanied by a decrease in ATP concentration and its enhanced breakdown, which was confirmed by the accumulation of ADP and AMP and indicates an imbalance between the rate of ATP formation and the pumping of ions, enhanced by an increase in Na^+^ influx into the RBCs of AD patients. In general, this means that constant and multiple activation of the enzyme in the RBCs of older AD patients experiencing restrictions in adapting to changes cannot completely compensate for the increased plasma membrane permeability that leads to negative consequences in chronic conditions [33,309], including enhancing the hydrolysis of ATP, 2,3-DPG, oxidative stress, and proteolysis [310]. The effect of this pathology, in combination with changes in the morphological and rheological properties, decreased the ability to deform in elderly RBCs [140,311], promoting dysfunction of RBCs mediated by a prolonged phase of deficient oxygen supply [140,312], thereby lowering the threshold for development of neuropathology [33,61,72,162,313]. Considering that the age of AD [314] and COVID-19 patients [20] was different and was somewhat heterogeneous in the COVID-19 group (mean age 58.4 ± 20.9 and 75 ± 2.6 for COVID-19 and AD patients, respectively), it is likely that the appearance of multidirectional changes in ATP and 2,3-DPG with a simultaneous increase in glycolysis in the RBCs of COVID-19 and AD patients reflects different age-related adaptive responses both to hypoxic conditions [315,316] and to different types of cytotoxic agents, although other factors cannot be excluded [317,318,319,320]. Considering the similarity of disturbances in the structure, morphology, energy metabolism, and antioxidant system of RBCs in these two diseases, it is therefore conceivable that cognitive impairment associated with hypoxia may have similar mechanisms. Further studies are needed to investigate this relationship and to confirm the clinical significance of the biomarkers of “RBC illness” as among the main components of a progressive and irreversible insufficiency of the integrated oxygen-transport system. These data may well hold the keys to understanding the pathogenesis and progression of irreversible brain damage and the permanent impairment of cognition that may significantly aggravate the course of severe systemic disease such as COVID-19 [321,322].

## 6. The Underappreciated Role of Erythrocytes in COVID-19

SARS-CoV-2 infection has spread rapidly around the world, but despite the collaborative efforts of the scientific community and clinicians there is to date no consensus regarding the pathogenesis of the disease, and there remains no effective antiviral therapy for COVID-19. A meta-analysis based on the findings of fundamental and clinical research provided evidence of strong correlation between numerous inflammatory markers, especially C-reactive protein (CRP), interleukin-6 (IL-6), ESR, and severity of COVID-19 [323,324,325,326]. This confirmed a suggestion that hyperinflammation (cytokine storm) is a basic pathologic mechanism of life-threatening multiple organ dysfunction caused by direct cytokine-induced tissue damage [8,9,10,11,12,13,14].

Surprisingly, RBCs are not included in the list of “affected” areas although they are also damaged during hyperinflammation [288,327,328,329], and lose their function [327,328] as oxygen carriers [330] and as “organs” impacting systemic metabolic homeostasis [81], and this predictable pathological relationship is not a tool for diagnosing “disease” relating to the RBCs themselves. It is noteworthy that IL-6 acts on a large number of cells and tissues [331] and, especially importantly, interacts with the RBCs [332] causing redistribution of membrane proteins and phospholipids [75], morphological changes, impaired deformability, increased oxidative stress [333], and accelerated cell aging [334], which altogether might be an important driving force in the development and progression of oxygen-transport dysfunction [322,324,331,335,336,337]. In light of this, we believe that underestimated metabolic and structural abnormalities of RBCs in COVID-19 patients should be an important area for future systematic research.

In addition, an increased ESR value obtained as a result of neutralization of negative charges on the surface of RBCs by acute phase proteins circulating in the blood during the inflammatory process [338] is exclusively perceived as an inflammation marker (although non-specific) [339]. The abnormalities of the RBCs themselves are not considered in clinical practice, although these may be associated with the harmful consequences of a decrease in charge on their surface [207,340], affecting not only their functional abilities but also the rate of capillary blood flow [341], the state of endothelial surface of blood vessels (negatively charged) [338,342,343], and in general the rheological properties of the blood.

It is obvious that the study of the state of RBCs and early diagnosis of their changes during the inflammatory process is very important for analyzing the state of blood flow in the microcirculation, which in turn largely depends on the ability of RBCs to deform [344]. It is essential to understand these data, especially considering that measurement of arterial oxygen saturation (SpO_2_) by the most common method of pulse oximetry under conditions of low blood flow and elevated carboxyhemoglobin and metHb levels [345] can lead to misinterpretation of gas exchange between the blood and tissues, delaying correct diagnosis and timely initiation of therapeutic interventions [346,347]. Therefore, we suggest that if the erythrocytes of COVID-19 patients contain increased amounts of abnormal forms of hemoglobin [281,348], these patients should be monitored using alternative indicators to avoid false results. Keeping in mind that the relationship between RBCs’ biochemical aberrations and their transport efficacy is a critical missing link in the understanding of life-threatening hypoxemia/hypoxia and multiple organ dysfunction during infection [233], we believe that the biomarkers of metabolic, antioxidant status disorders must be evaluated, including morphological and structural changes of the RBCs that form part of the integrated oxygen-transport system [18,19,20,21].

Finally, functional neuro-imaging studies of patients in different COVID-19 phases have shown that oxygen desaturation significantly influences the degree of brain hypometabolism and related cognitive deterioration [349,350]. Just as importantly, similar patterns of brain metabolic dysfunction have been identified in pediatric patients [351], supporting the accepted contemporary view that cerebral hypoperfusion and disturbed RBC metabolism and function are independent of the specific cause of the disease, and the patient’s age is the main trigger leading to the development of an energy crisis in the brain, neurodegeneration, and cognitive deficit [244,352,353], similar to those found in Alzheimer’s disease.

## 7. Conclusions

SARS-CoV-2 infection has spread rapidly around the world, but despite collaborative efforts of the scientific community and clinicians there is no consensus to date regarding the pathogenesis of the disease and remains no effective antiviral therapy for COVID-19. In addition to attacks on the respiratory system, Alzheimer’s-related symptoms have been widely reported in patients with COVID-19 and in those who have recovered from it. The mechanisms of brain pathology leading to cognitive dysfunction in COVID-19 patients are still not fully understood, and are multiple, but hypoxia-related brain pathology appears to be the main trigger involved in this disease. RBCs comprise one of the components in the integrated gas-transport system and are the only cells that carry oxygen to tissues, thus ensuring the vital activity of the whole organism. While researchers have long recognized the potential links between metabolic RBC aberration and hypoxia-related multiorgan failure, including the brain, the efficacy of oxygen delivery by RBCs is rarely assessed in combination with the other indicators usually measured in clinical settings to detect limited O_2_ transport.

Given the importance of addressing the development of functional disturbance of oxygen transport in RBCs, we suggest that a thorough and detailed study of erythrocyte metabolism and morphology is needed, not only to identify potential risk factors but also to shed light on the molecular mechanisms that limit oxygen supply to the tissues. This knowledge will be important to prevent the development of hypoxia-induced multiorgan failure and neurodegenerative processes, typical not only of AD but also of many other diseases with different etiologies, and characterized by varying degrees of cognitive dysfunctions, especially in COVID-19.

The development of technologies to assist in revascularization and restoration of erythrocyte metabolism must form an integral part of new therapeutic strategies in the treatment of a great variety of disorders associated with inadequate oxygen delivery, including devastating illnesses of human beings such as COVID-19.

## Data Availability

Data are contained within this article.
